# Do Animals Have Rights?

**DOI:** 10.3390/ani13071220

**Published:** 2023-03-31

**Authors:** Bernd Ladwig

**Affiliations:** Center for Political Theory and Philosophy, Otto-Suhr-Institute of Political Science, Freie Universität Berlin, Ihnestr. 22, 14195 Berlin, Germany; poltheorie@polsoz.fu-berlin.de

**Keywords:** animals, rights, morality, social justice, political membership

## Abstract

**Simple Summary:**

Sentient animals have moral rights. This follows from the best justification for human rights that we can give. However, that does not mean that animals have the same rights as we do. First, they have partially different interests. Second, humans have special relationships with each other, from which special duties also follow. Humans live in states and are subject to compulsory laws. However, we have also made many animals existentially dependent on us through subjugation. A just coexistence with such animals is only possible if we also grant them political membership rights.

**Abstract:**

Do animals have moral rights? An affirmative answer follows from the best justification for human rights that we can give. The moral status not only of humans but also of animals consists in an egalitarian right to have rights. From this equal status, however, substantially equal rights follow only if the morally relevant interests are equal. A reasonably broad and differentiated understanding of our own, human animal nature reveals which interests we share with many other animals. Thus, sentient animals have basic rights to life and well-being, including volitional activities and access to beneficial social relationships. Further rights arise from special human–animal relationships that are also politically relevant. By subjecting animals and, thus, making them existentially dependent on us, we owe them more than mere protection and help in easily remediable emergencies. We thereby also assume associative duties, as they exist among fellow citizens. Therefore, we should open our understanding of the common good to the reality of species-mixed communities and represent animals politically.

## 1. Introduction

All humans have rights understood as morally valid claims that are important enough to deserve legal protection. Due to their moral justification, no state is legitimate that systematically disregards them. Do some or all other animals also have rights in this particularly strong sense? Although in more recent times some legal scholars have argued for legal animal rights [1,2,3], the prevailing view among lawyers is that animals lack legal personhood. Consequently, most defenders of animal rights concentrate on the possible moral foundations in order to show that animals also deserve the strong legal protection that humans already enjoy.

The text is organized as follows. First, I outline the function and structure of rights. Then I sketch a model of moral reasoning in which we play the dual role of subjects and objects of moral consideration. Although animals are not capable of grasping the content of moral reasons, they share with us morally significant characteristics in which they basically deserve equal concern. Contrary to the prejudice that animals cannot have rights, I then show that at least the protective function of rights can also apply to them. All sentient animals have basic rights to life and well-being, including volitional activities and access to beneficial social relationships. Further rights arise from special human–animal relationships that are also politically relevant. Not all, but some animals thus have a valid claim to membership in a political community.

## 2. The Concept and Justification of Moral Rights

Morality is about duties. Not all, but central duties arise from rights. They follow from the valid claims of others. Whoever violates them does not simply act wrongly; they act wrongly by doing a wrong to others. They violate directed duties whose fulfillment would be owed to some other party [4]. Certainly, rights in this sense are not metaphysical dowries. They are not natural or metaphysical properties of individuals, as the natural law rhetoric of ‘rights inherent to all human beings’ might have us believe. This rhetoric would be better understood to mean that our fundamental rights do not first come into the world through political or social acts of bestowal. Rather, they emerge from moral reasoning itself.

A moral right is a special case of valid claims. It is a claim backed up by reasons that can be shared by all normatively responsible persons. I presuppose that at least human rights satisfy this condition. Since human rights, unlike animal rights, are in principle not very controversial, they shall serve me as a model case for the following conceptual explication. I will methodically progress from the more familiar to the less familiar by showing that from the best understanding of human rights claims it follows that many animals also have moral rights [5].

Claims always have a three-digit structure. A (a subject) has a claim to X (a good) against B (a bearer of the duty or obligation). The relation can also be logically reversed: B has a duty with respect to X against A. This fact supports the so-called correlativity thesis: no rights of one party without substantively corresponding duties of another [6]. This is not entirely wrong, but it must not obscure an essential asymmetry: Claim rights give rise to the duties that correspond to them. B is obligated to do or omit something because A has the practical authority [7] to demand this of B. B owes A the fulfillment of the obligation. The normative relationship emanates from A, the holder of the right [8]. The latter may directly and for their own sake expect B to do or not to do something. Without this practical authority embodied by A, the duty would not exist.

Human rights are a subclass of claim rights. They are morally justified claims that every human being possesses as such. A minimal consensus states that every born and not (entirely) brain-dead individual member of the species Homo Sapiens is necessarily and inalienably endowed with certain rights, including the rights to life and physical integrity. Normatively, they apply even if the positive law of a state does not provide for them. The convincing moral justification, not the actual legal implementation and observance, constitutes the condition of the existence of human rights. We react specifically morally, for example with indignation [9], where we believe them to have been violated; and our indignation would not diminish if we learned that those in power did not believe in human rights and that the laws of the state therefore did not entail them. Rather, we would see this as an additional reason for moral resentment.

What might a convincing moral justification for human rights look like? Let us imagine a moral discourse about what we owe to each other as human beings. Such a discourse has a practical purpose: it aims at reasons for norms of action that all normatively responsible persons should see as categorically binding. Every sane person should incorporate a validly grounded norm of morality into their conscience. The reasons that speak for the validity of the norm must therefore not only be valid relative to particular social positions. They must be acceptable for all independent of their bargaining power and other properties that separate agents from each other. Valid norms of morality are based on reasons all moral agents can share [10].

However, human persons are not only addressees of moral responsibility. They also require moral consideration from all other moral actors. They are not only moral agents, but also moral patients. We should therefore presuppose that any participant in moral discourse also regards themselves as a vulnerable being worthy of consideration. Each of them has their own point of view of the world, makes their own experiences, and is irreplaceable in the conduct of their life, the success of which is of ultimate value for any of them. Any agent will therefore want to reconcile the norms of morality with the pursuit of a successful life. In Kantian terms: Any moral agent sees themselves as an end and never merely as a means [11].

This rules out a purely aggregative view of morality, as represented by utilitarianism. Any moral agent will reasonably attach importance to not letting their most important interests fall under the wheels of collective goal pursuit. We will all demand guarantees that our individual good cannot simply be sacrificed for a supra-personal overall good. The moral status therefore consists in a right to have rights [5]. This concept, redundant only in appearance, emphasizes the difference from a purely aggregative moral conception. Moral agents not only have the right to appear with their utility functions in an ultimately impersonal moral calculus. The right to have rights gives each member of the moral community the normative authority to demand from all others a consideration owed to him or her. Otherwise, a normative order would not merit the consent of every participant in a moral discourse. The normative order must be characterized by the form of subjective rights. In addition, since the participants in the moral discourse are all human beings, they will grant each other rights as human beings.

## 3. Rights beyond Anthropocentrism

The picture that emerges is apparently quite anthropocentric. This is true of the criterion of universal consent and equally true of the circle of rights holders that results from it. However, this is simply because I have started from a discourse in which moral agents seek to work out together what they owe to each other generally and reciprocally. Since only human persons can recognize and critically review moral duties as such, there is no alternative to an anthropocentrism of morality in this epistemological respect. However, a genuinely normative question distinct from this is who should count as a moral patient. Who belongs to the community of beings who deserve consideration for their own sake in the form of subjective rights?

To answer this second question, participants in the discourse must refer to and reflect upon their own moral neediness as vulnerable individuals. They should ask themselves what aspects of their vulnerability give them valid reasons for claiming consideration in the form of human rights. In doing so, they should not be distracted by who else might benefit from the consideration owed to them. This follows from the formal generality of morality. It requires us to give equal consideration to the same morally significant properties in otherwise equal circumstances.

Only the content of a valid justifying reason therefore determines the condition of moral belonging in the respect specified by this very reason, and only the facts decide who fulfills the condition of belonging. If respect is due to us because of our disposition to autonomy, then in that regard only individuals belong to it who are at least potentially normatively sane and morally responsible. With regard to the sensitivity to pain, on the other hand, all who can feel pain like we do belong to the community of beings to whom no unnecessary pain may be inflicted. After all, at least one reason that we (should) consider morally significant applies to them as well as to us [12].

This suggests a pluralistic picture of interests relevant to human rights [3]. It is not determined solely by the higher capacities that distinguish us as normatively sane persons and morally capable actors. Certainly, such specifically human capacities play a role in making some human rights intelligible. Only those who can reason and articulate linguistically have something to gain directly from the freedom of linguistic expression. Only those who can understand what offices and authorizing acts are can meaningfully exercise a political right to vote. Only those who can understand their own moral status have to worry about their self-respect. Even violations such as torture are also abhorrent in human rights terms because the victims are humiliated by them, and their will as autonomous beings is to be broken.

However, that is hardly all that makes torture terrible and utterly reprehensible. Shall we give no weight of its own to the aspect of body-bound torment? After all, severe pain is bad in itself. It is so regardless of whether the victim is a brilliant intellectual or a one-year-old child, whether they can speak or only scream. Torture tends to reduce even mature people to mere creatures trembling for their lives and writhing in pain, and it makes them experience in an extreme way that they are creatures capable of suffering.

Perhaps we suffer pain in a special way because we can also ask the question about its meaning and possible justification. In addition, we certainly do not interpret every intentional infliction of pain, no matter how severe, as torture. The interpretation of an infliction of suffering as torture implies the accusation that the procedure is at least prima facie wrong. However, it would certainly also be wrong to pull out the nails of a newborn child without compelling reasons. Moreover, it would be absurd to assume that the only decisive factor for the type and severity of the pain is whether the victim is aware of the injustice that he or she may experience through the treatment.

Thus, although we started from human persons as participants in moral discourses, interests also become recognizable as relevant to human rights, which the participants have in common with many other creatures. Not only human beings who can relate to reasons as reasons and can obey duties out of insight can have a more or less good or even only bearable life. Immature and severely mentally impaired people can also experience their existence as more or less gratifying, and the same is true for many other animals. They all possess not only physical but also psychological characteristics that grant them their own perspective on their existence in the world. They are all irreplaceable individual subjects experiencing, or suffering, some of their life processes. This ontological feature separates humans and all sentient animals from inanimate objects, but equally, as far as we know, from simple animals such as nematodes, from all plants, fungi, and microorganisms [13].

This is also normatively relevant because all sentient beings possess interests that are eligible for morally owed protection and morally demanded promotion. In our own case, this protection and promotion take the form of human rights. Yet, we share some interests relevant to human rights in this way or in a similar way with many other animals capable of feeling and experiencing. After all, we are not only rational, linguistically gifted, and moral beings, but also bodily existing finite creatures that can suffer and need bonding. We should therefore also grant morally justified rights to all other such creatures. They share with us the fact that their lives matter to them subjectively and that the success of their lives is of ultimate value to them. Thus, they should all be recognized as ends and never merely as means [14].

It is worth noting that the argument sketched out so far is not identical to the notorious argument from human marginal cases (AHMC) [15]. The AHMC also starts with the basic moral imperative to treat like cases alike. We establish the rights of humans based on characteristics that we consider morally significant. We share some of these characteristics with many other animals. If we nevertheless exclude them or put them in a worse position, it seems to be only because of their different genetic makeup. Alternatively, we could try to base human rights solely on such properties that no other animal shares with us. This counts in favor of recourse to our ‘higher’ capacities such as linguistic agency and normative sanity. If only these higher abilities were relevant to our rights, we would indeed be allowed to exclude all other animals.

Unfortunately, however, we would thereby also exclude many fellow human beings. Some born and not (completely) brain-dead members of our species are not even potentially capable of propositional language and normative sanity because they are, for example, severely mentally handicapped from birth. Do they therefore have no human rights? Those who consider this counterintuitive and downright abhorrent, however, seemingly have to concede that some nonhuman animals have the fundamentally same rights as comparably competent—or impaired—humans do. In short, our ‘higher’ traits may constitute human monopolies, but not all humans possess them. Such properties, on the other hand, which are possessed by just about all born and non (whole) brain dead humans, are not human monopolies; we share them with many other animals. Thus, we need to include these other animals as well. This simple ‘moral set theory’ is the essence of the AHMC.

However, my argument presented above is not dependent on the truth of the AHMC. It does not refer specifically to mentally impaired people. Instead, it relies on an appropriately broad understanding of the animal nature of even all mature human beings. Such an understanding counts in favor of a pluralistic conception of the morally relevant interests of humans. It thus simultaneously forms a bridge across the species barrier. It allows us to decide, free of arbitrariness, which interests of animals also morally deserve protection and promotion. Many animals possess interests that we among us humans consider sufficient to protect and promote them by means of moral rights. Accordingly, it follows from the basic moral precept of equal treatment of equal cases that an animal possesses a right in precisely that respect in which it sufficiently resembles a human being who possesses a right in this very regard.

A possible objection to this last claim is that it is inappropriately anthropocentric. It might suggest that the more an animal resembles us, humans, the more it is morally worthy of protection and promotion. However, an interest does not have to be the same in all respects for all people and all animals in terms of content. Different individual animals of different species have different needs and abilities and can therefore be hurt in different ways [13]. For example, sensory deprivation means something different for an animal that orientates itself primarily by means of the sense of smell than it does for an animal that uses an echo sounder.

Once we recognize that we share a basic dimension of interest with other animals, we should be open to morally relevant differences within that dimension. Thus, it is only the epistemic starting point of morality which is necessarily anthropocentric: in moral judgments, we cannot help but begin with a substantive respect in which we owe something to each other in order to take the step across the species barrier from there. Without any similarities to us, we simply could not decide whether and with regard to what we should consider an animal. However, this does not force us to anthropomorphize those animal interests, which we in this way reveal as morally relevant.

## 4. Objections to the Possibility of Animal Rights

Notwithstanding the partial overlap of morally relevant interests, some philosophers and lawyers argue that animals cannot have rights. Three such arguments should be considered. A first one starts with the correlativity of rights and duties. Almost all animals except humans are incapable of insightful compliance with the rights of others. They lack the ability to accept an obligation from insight into the validity of the reasons justifying it. However, the view that only those who can observe moral obligations arising from rights can have rights themselves is already implausible with regard to human rights. Even small children or people with severe dementia cannot insightfully respect other people’s rights.

A second argument refers to a distinction drawn by the legal philosopher Joel Feinberg [16]. Those who confidently make use of their normative possibilities as rights holders thus testify to their self-respect. By claiming rights, they can value themselves as holders of valid claims. Feinberg argues that this special self-relation is only conceivable in a world with rights. He illustrates this with the counter-image of a world only with duties. In this counter-world—Feinberg calls it Nowheresville—a considerate interaction on all sides would probably be possible. Perhaps no one would have to fear for their life, their health, or their basic freedoms. Perhaps everyone would treat each other nicely. However, no one could say that certain actions were owed to her. No one could demand that others do or refrain from doing something for her sake. In this important respect, Nowheresville would remain normatively deficient: it would be a moral world without self-respecting subjects.

However, if rights enrich the moral universe by enabling self-respect, does this not confirm the thesis that only self-conscious subjects can have rights? After all, only they have a self-respect to gain or lose. Animals cannot conceptualize themselves as rights-bearers and cannot derive any self-respect from this status. There is a conceptual reason, however, why enabling self-respect cannot be the very ground of rights. Self-respect is not a good that we are entitled to because we desperately need it. It is a genuinely normative good. Those who respect themselves thereby testify that they know about and affirm their own moral status as subjects of rights.

However, this means that the commanded respect logically precedes self-respect. Nobody has a claim to respect herself for something which is not already due to her independently of this self-relation. The fundamental claim can therefore only be the claim to respect itself. Self-respect is systematically secondary to it. This does not invalidate Feinberg’s insight that normative orders with rights, unlike those that entail only duties or even virtues, produce subjects who can respect themselves. This is an additional advantage of rights-oriented orders, but it does not give rise to the moral status on which self-respect is based.

Feinberg himself denied that only possible subjects of self-respect could also be subjects of rights. Even though rights can be claimed, the person who claims something and the individual in whose name she claims it do not have to be identical with each other. According to Feinberg, every individual with their own interests can be considered a right holder. That applies to young children and likewise to many animals [17]. Others could exercise the claims as proxy representatives for them.

However, does this not mean that something essential is lost? A merely advocatory use of rights may be conceptually possible, but it seems to be part of the special value of rights that they give us possibilities for the self-confident shaping of normative relationships. The connection between rights and self-respect points to the emancipatory value of valid claims. This added value of rights is emphasized by supporters of the will theory, which, as the name already suggests, accentuates the willpower of the rights holder [18]. The latter can use their rights as they see fit and thereby also change normative relationships. Thus, an owner can reclaim a loan, appeal to the courts, or even make others into owners with their consent. Holders of rights have freedoms and powers that they would not possess in a world with only duties or even virtues. Subjective rights in the sense of will theory are thus an important embodiment of the modern basic value of personal self-determination or autonomy.

From the will theory emerges the third reason why animals could not have rights. Almost all of them, after all, are incapable of self-conscious and self-determined use of claims. They possess at best a rudimentary concept of moral claims and normative relations as such. However, do rights really always include freedoms such as the use of a thing at one’s own discretion and powers such as to transfer property? There are two reasons against such a generalization [19].

First, the will theory leaves no room for inalienable rights. An inalienable right is a valid claim that the holder may not relinquish or cede to others. Thus, the right not to be enslaved arguably includes the prohibition against offering oneself for enslavement. Consent to an enslavement contract would be normatively void: no one would possess the competence to enter into such a contract. Similarly, procedural rights such as the right to a fair trial are not primarily there to bring the free will of the accused to bear. The latter may have the freedom to choose a defense counsel or a particular defense strategy, but she does not therefore also have the freedom to forego overall fair treatment in court. Second, the will theory has a socially exclusive effect. It misses all subjects who are not yet, no longer, or never will be capable of self-conscious self-determination. This is not a marginal problem, and it does not only concern animals. It affects all human beings in the early, and many in the later stages of their lives. Once again, the solution lies in the possibility of using rights by proxy.

The will theory is therefore not a good general framework for a conception of claim rights. It tells us how self-aware subjects can use some of their rights, but it tells us too little about what rights might be good for. That is rather the domain of the interest theory. The latter emphasizes the substantial benefits that rights offer us. According to the interest theory, an individual X can have rights if some aspect of X’s well-being (an interest) is weighty enough for holding some moral agents under a duty [20]. Sentient animals are capable of subjective well-being and can therefore be considered rights bearers in the sense of the interest theory.

To be sure, animal rights lack the kind of emancipatory added value that will theorists emphasize. Their central purpose is protection, not personal autonomy; however, the protective function of the rights is essential, especially for animals. Today, many of them are even excluded by definition from the space of rights-bearers. They are labeled as ‘livestock’ or as ‘pests’ and thus reduced to possible contributions to the fulfillment or thwarting of human purposes. Likewise, humans dispose of animal habitats without appreciably considering their importance to the well-being and flourishing of untold numbers of animals. Humans decide on new settlements, roads, power lines, cultivation areas, or spoil heaps and regard animal territories as terra nullius [21].

In the meantime, many states have enacted laws to protect animals. German animal protection law, for example, stipulates that animals must be stunned before slaughter. However, the law basically serves to regulate the use and also killing of animals for foreign, primarily human purposes. The so-called farm animals are only in the world to be used or consumed by humans. They have no independent right to their own life. Our interests of use form the framework of their consideration by animal protection norms. These norms should ease the animals’ lot after it is already determined that they should give us meat, milk, eggs, leather, or other products and may be locked up and slaughtered for it.

However, sentient animals are not our natural servants. They are individuals with a life of their own, which for each of them is the only one they have. To recognize them as holders of rights would mean to respect them as ends instead of seeing them merely as means. In addition, even if very few rights apply absolutely, they do imply strict justification obligations for any violation of basic goods [22]. Most animals would objectively benefit greatly if at least their painful and life-shortening use for comparatively trivial purposes such as meat-eating were excluded and if they were no longer allowed to be mere material for medical experiments. Animals would even benefit from rights in a special way: they are particularly vulnerable inhabitants of a world designed by humans for humans, they cannot defend their interests themselves through collective action, and the vast majority of them cannot hope for our spontaneous sympathy, as dogs, cats, or canaries might.

## 5. What Rights Do Animals Have?

In order to have a morally relevant interest, an animal must only be sentient. Sensations are not value-neutral, for they have a more or less pronounced positive or negative valence such as joy or pain. In addition, sentient animals actively deal with their environment and care about their own well-being and survival in at least a behavioral sense. Therefore, we should not only regard them as sentient but also as capable of experiencing in a broader sense, including strivings and volitions [13].

We can therefore give a broad outline of the content of animals’ moral claims: All sentient animals capable of experience have at least interests in the dimension of well-being, which includes pleasant sensations and pleasurable experiences as well as scope for volitional activities. Negatively, this means we must neither let animals suffer without necessity nor prevent them from engaging in activities that are promising for them. In addition, as far as they are social beings, like all animals domesticated by us, they must be able to establish contact with conspecifics or other—human or non-human—animals in forms that fit their social dispositions. I think, moreover, what I can only assert here, that we should also grant animals capable of experiencing a fundamental interest in their own further life. After all, life is a condition of the possibility of any beneficial experiences and activities, and we should not deprive animals of this possibility without necessity by killing them.

Last but not least, we also know how strong animal legal claims are in principle: they are just as strong as the claims of any human being based on similar interests. This, in turn, follows from the basic requirement of equal treatment of equal cases: equal morally relevant interests count alike, no matter whose interests they are. In this sense, all the bearers of moral rights possess fundamentally the same status irrespective of species affiliation.

However, it does not follow from this that all animals that have any moral rights at all, therefore have the same rights as humans do [22]. The assumption of a fundamental equality of status is, first, compatible with the fact that animals do not have certain rights that humans have, because they do not bring them any advantages. Incidentally, this is again already true among humans. A political right to vote for very young children makes no more sense than a right to vote for foxes. Animals may benefit from how people exercise their right to vote who can exercise it intelligently, but they are then only indirect beneficiaries of a right that they themselves could not use with rhyme and reason.

Rights to political and also personal autonomy are, generally speaking, only directly significant for individuals who can bear a normative responsibility for living together and for leading their own lives. For the vast majority of non-human animals, this does not apply even in a rudimentary sense. Likewise, protection against discrimination or symbolic degradation is at most indirectly significant for them, since they cannot understand their own valid claims as such and relate them to the valid claims of others. This means, for example, that animals cannot suffer as such from the fact that humans somehow dominate them [23]. In fact, it seems to me that human animal husbandry is permissible precisely when it is consistent with the principle of equal regard for all the interests that animals actually have: in continued life, in well-being, and also in volitional activities and access to beneficial social relationships. Conversely, however, this means that almost all commercial and, without exception, industrial animal husbandry is incompatible with this principle and should therefore be abolished.

A second type of differentiation is permissible where animals have similar morally significant interests as we do, but these interests are less strong in their case. This is certainly the more complicated case, and we must not conclude from the fact that, e.g., a restriction of freedom of movement affects an animal and not a human being that it is therefore less severe. Nevertheless, some restrictions will affect a more complex experiencing animal more strongly and in more dimensions than a less complex experiencing animal, and human persons are the most complex animals we know.

Maybe we should therefore also assume that human persons, or self-conscious subjects in general, have a uniquely strong interest in their own survival. After all, they can refer to their own existence in a forward-looking or also in a retrospective way, wish for its continuation, and pursue longer-term projects, which only consciously but not self-consciously living animals are not able to do. To argue for such a position on the gradability of the interest in life would lead us into our very own philosophical depths [24]. Here it may suffice to point out that the presence of an interest across species does not necessarily mean that it is present everywhere in the same strength. Moreover, to consider unequal interests unequally would not be arbitrary in itself. It would certainly be arbitrary, however, if we killed an animal for a minor advantage such as the pleasure of a palate. At least in societies like ours, all people could exist well and healthy without products for which animals live miserably and die violently.

## 6. Social Relations and Membership Rights

I arrived at this result of a moral status equality of humans and (other) animals by inserting the principle of equal consideration of interests into the framework of a conception of moral rights. However, the obligations of moral agents toward an individual do not sufficiently emerge from the latter’s moral status. Also relevant is the relationship between the duty bearers and the bearers of the rights. This consideration sets limits on the conceptional continuity between human and animal rights. Human rights are not only moral ‘possessions’ of individual human beings, but they also regulate social relations and should enable social participation and joint action.

As far as possible, all people would have to have, first, and fundamentally, institutionally secured access to as many human rights goods as possible [25]. Human rights therefore require more from us than just the renunciation of harmful behavior by moral actors, effective protection against such behavior, and reparation or compensation after such behavior. They require us to respond effectively, comprehensively, and even proactively to whatever threats to basic goods may arise. Consequently, we encounter human rights obligations on two different levels: on a directly goods-related level involving respect, protection, and assistance (if possible, for self-help); on a logically higher level as obligations to form or strengthen institutions in order to be able to fulfill obligations on the first level as effectively as possible.

To be sure, we also need institutions to prevent people from hunting animals in the wild or harming them in other ways, such as by destroying the environment. Animal rights then form a protective belt to shield animal habitats and life forms against the negative consequences of human intrusion and encroachment. However, humans are not the only beings and forces that threaten animals’ basic interests. Wild animals are also at risk of starvation, thirst, freezing, or being killed by other animals regardless of the consequences of human activities. If we wanted to effectively protect them from all these dangers, we would have to cover their habitats with institutions that would turn the wilderness into a kind of giant wildlife park. To be sure, virtually every wilderness today is already heavily infused and shaped by human activities [13]; and to the extent that we have harmed wildlife as a result, we owe them reparation or compensation [26]. However, compensation cannot reasonably consist of moralizing and transforming the last remnants of wilderness according to human conceptions. Insofar as wild animals are to remain wild, the human rights ideal of a humane and justly regulated world cannot apply to their habitats.

A second, related difference is that human rights, beyond their defensive and protective function, should secure the conditions of effective inclusion in political and social communities. They are meant to enable cooperative, solidary, and caring relationships among free and equal citizens. Some political philosophers even consider the membership function of human rights to be paramount [27]. However, once again, this finds no counterpart in our relationship with wild animals, as envisioned by most animal rights advocates. The prevailing view is that we should leave wild animals alone as far as possible and reasonable. Rights then have the primary function of protection from human encroachment [28]. At most, limited and predominantly interactional duties to help in emergencies are compatible with them.

This does not mean, however, that our relationship with wild animals must be paradigmatic for our relationship with animals in general. Representatives of contextualist [26] and political [5,21,29] theories of animal rights emphasize that we have brought many animals directly into our ways of life through breeding and husbandry. Many domesticated animals, in particular, could not stay alive, or at least not have good lives, without regular human attention. A possible alternative to letting such animals slowly but surely die out would be to recognize them as members of our communities. Their rights would then also acquire a membership function. It would include social, and in the case of working animals, also labor rights [30].

Anyone who keeps animals thus has extensive support obligations towards them. The animals have the right to active attention to their welfare through appropriate housing, nutrition, supervision, and care. In addition, we must all ensure, through our state, that the animal keepers fulfill their guarantor obligations. One might spontaneously find the idea of compulsory health insurance for domestic pigs or state-guaranteed pension insurance for guide dogs laughable. However, the only normatively acceptable alternative would be not to keep and use such animals as companions or cooperation partners in the first place. Once such animals are there, we cannot excuse ourselves by insisting that they should see to it themselves how they might cope in case of illness or in old age. After all, we have forced them, directly or through our legislation, into circumstances in which they could hardly care for themselves.

As citizens, we are responsible for the laws that permit the keeping and use of animals. This, in turn, speaks to a socially expanded understanding of the common good. We should make political decisions with the awareness that the collective for which they claim binding force includes humans and animals. For such a species-mixed community, what Ronald Dworkin understands as the sovereign virtue of political morality must apply: no state is legitimate that does not pay fundamentally equal consideration to the fortunes of all its members [31].

Although Dworkin limits this basic norm to fellow human citizens, we should apply it to all animals we subject through our institutional orders. We owe these animals the fulfillment of duties of social justice. The basic social order regulated by law and armed with coercion must be acceptable from the perspective of really all individuals who belong to it or are existentially dependent upon it. Each individual must be able to reconcile the norms that regulate coexistence with their fundamental claim to live their own life, according to their basic needs and capabilities. This fundamental claim is also possessed by animals that cannot take a stand on moral and political norms themselves. Every norm that concerns them must also be acceptable from their perspective. This follows from the extension of an all-subjected principle to human–animal relations.

I therefore propose a political membership status for all animals whose living conditions we comprehensively control and who could not live well or at all without regular human attention. It would include the right to live on a state territory and to return to it. Likewise, the interests of animals should receive fundamentally equal consideration in determining the common good [21]. To this end, animals require representation by human proxies in the political process. The consideration of animal interests is a cross-cutting political task that should be anchored at the macro-level of political decision-making.

However, how can we know which of the animals’ interests their human representatives should advocate for? The general answer is that animals could give us valuable clues about their needs and preferences through their own activity [21]. Many animals can communicate with us nonverbally. They can tell us what is important for their well-being. For this reason, animal members should have the right to become visible in our midst and to develop as unhindered as possible and feasible. For the same reasons, animals should also be allowed to express their own will in working relationships. We must first respect the fact that they have a will that we may influence, through offers and incentives, but which we must not break. Secondly, we must learn to interpret the animals’ expressions of will with a view to possible consequences for their welfare. Thirdly, we must offer them possibilities of evasion and refusal without penalty. Subjecting animals to an alien purpose against their stable will is a tyrannical form of exploitation.

## 7. Conclusions

The moral status not only of humans but also of animals consists in an egalitarian right to have rights. From this equal status, however, substantially equal rights follow only if the morally relevant interests are equal. We do not need to resort to the argument from human marginal cases for this purpose. Already a reasonably broad and differentiated understanding of our own, human animal nature reveals which interests we share with many other animals. These, too, possess interests in the dimensions of life, well-being, and volitional activities. By contrast, at least the vast majority of animals have no interest in autonomy and self-respect.

I consider justly regulated lasting relationships with some animals to be possible and also desirable. However, by subjecting animals and, thus, making them existentially dependent on us, we owe them more than just protection and help in easily remediable emergencies. We thereby also assume associative duties, as they exist among fellow citizens. Therefore, we should open our understanding of the common good to the reality of species-mixed communities and represent animals politically. However, we should essentially let wild animals be wild, even if this means that we restrain ourselves in our capacity to intervene.

## Data Availability

Data sharing is not applicable.
